# Transition Into Clinical Routine and Survival Outcomes of BCL2‐ and BTK Inhibitors: An Analysis of Patient Data From the GCLLSG Registry

**DOI:** 10.1111/ejh.70178

**Published:** 2026-04-14

**Authors:** Nadine Kutsch, Rudy Ligtvoet, Sandra Robrecht, Hartmut Linde, Thomas Illmer, Steffen Dörfel, Jörg Lipke, Ali Aldaoud, Rudolf Schlag, Jolanta Dengler, Othman Al‐Sawaf, Petra Langerbeins, Paula Cramer, Michael Hallek, Barbara Eichhorst, Kirsten Fischer, Anna Fink

**Affiliations:** ^1^ Department I of Internal Medicine, Center for Integrated Oncology Aachen Bonn Cologne Duesseldorf, German CLL Study Group University of Cologne Cologne Germany; ^2^ Practice for Blood and Cancer Diseases Potsdam Germany; ^3^ Practice for Hematology and Oncology Dresden Germany; ^4^ Oncozentrum Dresden, Freiberg, Meißen Germany; ^5^ Practice for Hematology and Oncology Dortmund Germany; ^6^ Practice for Hematology and Oncology Würzburg Germany; ^7^ Practice for Oncology Heilbronn Germany

## Abstract

Within a dataset of the German CLL Study Group (GCLLSG) registry, 274 patients were allocated to a cohort with venetoclax and 888 to a cohort with BTKi (79 acalabrutinib, 809 ibrutinib), each as first administered targeted substance class within the documented treatment sequence. Venetoclax was administered as first‐line treatment in 152 of 274 patients (55.5%). After a median observation time of 14 months from the start of the first documented venetoclax treatment, the 1‐year event‐free survival (EFS) was 82.1% and the 1‐year overall survival (OS) was 94.2%. The 1‐year OS was 96.3% when venetoclax was given as first‐line treatment versus 92.1% when given for the first time in a later therapy line (HR 0.38, 95% CI 0.14–1.05). In the BTKi cohort, 351 of 888 patients (39.5%) received a BTKi as first‐line treatment. The median observation time was 34 months from the start of the first documented BTKi treatment, with a 3‐year EFS of 32.4% and a 3‐year OS of 78.5%. The 3‐year OS for a BTKi in first‐line was 87.9% compared to 73.8% in a later therapy line (HR 0.43, 95% CI 0.30–0.61). Our analysis suggests better survival outcomes when these agents are used as first‐line therapy with the caveat that patients treated in later therapy lines typically exhibit more advanced disease and a poorer general condition.

## 1 Introduction

BCL2‐inhibitors (BCL2i) and covalent BTK‐inhibitors (BTKi) have profoundly improved the outcome of patients with chronic lymphocytic leukemia (CLL) in the last decade [1, 2, 3, 4, 5, 6, 7, 8] and are considered standard treatment [9, 10]. However, the transition from treatments evaluated within clinical trials into routine clinical practice can be complex. We present data from the German CLL Study Group (GCLLSG) registry of two patient cohorts treated with either BCL2i (venetoclax) or BTKi (acalabrutinib or ibrutinib) as first administered targeted substance class in the documented treatment sequence, with the aim to analyze patient characteristics, transition into clinical routine with previous and subsequent treatments, as well as efficacy and survival outcomes.

Patients from multiple German sites with confirmed diagnosis of CLL, who have received first‐line CLL treatment between July 1st 2014 (i.e., after approval of ibrutinib for the treatment of CLL by the European Medicines Agency; EMA) and January 30th 2023 (data cut‐off date of this analysis) and who have received at least one documented CLL treatment with venetoclax or BTKi were included in this analysis. Patients who have been treated with venetoclax and BTKi in different lines of therapy were allocated to the venetoclax cohort if venetoclax was administered first, and to the BTKi cohort if BTKi was administered first. Patients participating in an active clinical trial of the GCLLSG were excluded and treatments were categorized according to the backbone of therapy. Time to next treatment (TTNT, defined as time until start of subsequent CLL treatment), event‐free survival (EFS, defined as time until start of subsequent CLL treatment, disease progression, or death) and overall survival (OS, defined as time until death) were analyzed using Kaplan–Meier methods.

A total of 1162 patients were included in this analysis, of which 274 patients were allocated to the venetoclax cohort and 888 patients to the BTKi cohort (79 acalabrutinib, 809 ibrutinib). Median observation time from first‐line treatment was 26 months (range 0–296) in the venetoclax cohort and 75 months (range 0–266) in the BTKi cohort, whereas the median observation time from first treatment with either venetoclax or BTKi, irrespective of therapy line, was 14 (range 0–97) and 34 months (range 0–120). The median time between first diagnosis and first venetoclax or BTKi treatment was 6 (range 0–26) and 6 years (range 0–37), respectively.

Venetoclax was administered as monotherapy in 70 patients (25.5%), in combination with an anti‐CD20 antibody in 203 patients (74.1%), and in combination with bendamustine in one patient (0.4%). In the BTKi cohort, 797 patients (89.8%) received a BTKi‐based monotherapy, 82 patients (9.2%) in combination with an anti‐CD20 antibody, and 9 patients (1.0%) received a BTKi in combination with another agent like chemotherapy or prednisone. At the time of analysis, treatment was ongoing in 94 of 274 venetoclax‐treated patients (34.3%) and in 434 of 888 BTKi‐treated patients (48.9%), and reasons for treatment discontinuation were available for 112 and 276 patients, respectively. Treatment was terminated as planned in 71 of 112 venetoclax‐treated patients (63.4%) as compared to 26 of 276 BTKi‐treated patients (9.4%). In most cases, premature treatment discontinuation was due to adverse events or intercurrent illness in 17.0% and 44.2%.

In the venetoclax cohort, median age at the time of first venetoclax treatment was 71 years (range 32–87) with 172 male patients (62.8%). Median cumulative illness rating scale (CIRS) score was 2 (range 0–13) and 12 patients (7.5%) had an ECOG performance status > 1. Among 57 patients with known molecular genetics, 32 patients (56.1%) had unmutated IGHV (uIGHV) and 9 out of 51 evaluable patients (17.6%) had a deletion and/or mutation *TP53*. Applying the CLL‐IPI, 11 out of 58 patients (19.0%) were in the very high‐risk group. Further patient characteristics are depicted in Table 1.

**TABLE 1:** Patient characteristics.

Patient characteristics by cohort	Ven	BTKi	Total
All patients	**274**	**888**	**1162**
Age (years)	**274**	**888**	**1162**
Median	71	72	72
Range	32–87	32–92	32–92
Age (years), *N* (%)	**274**	**888**	**1162**
≤ 65	93 (33.9)	270 (30.4)	363 (31.2)
Gender, *N* (%)	**274**	**888**	**1162**
Male	172 (62.8)	610 (68.7)	782 (67.3)
CIRS total score	**165**	**485**	**650**
Median	2	3	3
Range	0–13	0–17	0–17
CIRS total score, *N* (%)	**165**	**485**	**650**
> 6	27 (16.4)	80 (16.5)	107 (16.5)
*Missing*	*109 (39.8)*	*403 (45.4)*	*512 (44.1)*
CIRS organ system heart, *N* (%)	**164**	**475**	**639**
> 0	53 (32.3)	118 (24.8)	171 (26.8)
*Missing*	*110 (40.1)*	*413 (46.5)*	*523 (45.0)*
ECOG performance status, *N* (%)	**160**	**467**	**627**
= 2–3	12 (7.5)	45 (9.6)	57 (9.1)
*Missing*	*114 (41.6)*	*421 (47.4)*	*535 (46.0)*
Binet stage, *N* (%)	**137**	**481**	**618**
A	29 (21.2)	88 (18.3)	117 (18.9)
B	56 (40.9)	193 (40.1)	249 (40.3)
C	52 (38.0)	200 (41.6)	252 (40.8)
*Missing*	*137 (50.0)*	*407 (45.8)*	*544 (46.8)*
Cytogenetic subgroup, *N* (%)	**57**	**171**	**228**
Deletion 17p	7 (12.3)	42 (24.6)	49 (21.5)
Deletion 11q	7 (12.3)	21 (12.3)	28 (12.3)
Trisomy 12	4 (7.0)	19 (11.1)	23 (10.1)
No abnormalities	20 (35.1)	52 (30.4)	72 (31.6)
Deletion 13q	19 (33.3)	37 (21.6)	56 (24.6)
*Missing*	*217 (79.2)*	*717 (80.7)*	*934 (80.4)*
IGHV mutation status, *N* (%)	**57**	**156**	**213**
Unmutated	32 (56.1)	104 (66.7)	136 (63.8)
*Missing*	*217 (79.2)*	*732 (82.4)*	*949 (81.7)*
*TP53* mutation status, *N* (%)	**74**	**177**	**251**
Mutated	9 (12.2)	66 (37.3)	75 (29.9)
*Missing*	*200 (73.0)*	*711 (80.1)*	*911 (78.4)*
*TP53* status, *N* (%)	**51**	**130**	**181**
Deletion and/or mutation	9 (17.6)	61 (46.9)	70 (38.7)
*Missing*	*223 (81.4)*	*758 (85.4)*	*981 (84.4)*
CLL‐IPI score, *N* (%)	**58**	**142**	**200**
Low	4 (6.9)	3 (2.1)	7 (3.5)
Intermediate	12 (20.7)	24 (16.9)	36 (18.0)
High	31 (53.4)	69 (48.6)	98 (49.0)
Very high	11 (19.0)	46 (32.4)	59 (29.5)
*Missing*	*216 (78.8)*	*746 (84.0)*	*962 (82.8)*

**FIGURE 1:**
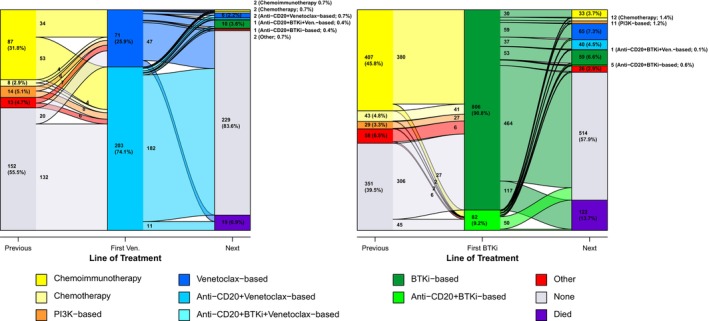
(a) Sankey plot of last treatments before first venetoclax regimen and first subsequent therapies. (b) Sankey plot of last treatments before first BTKi regimen and first subsequent therapies.

Most patients in the venetoclax cohort received no prior treatment before first administration of venetoclax (median number of prior treatments = 0; range 0–9), with venetoclax administered as first‐line treatment in 152 of 274 patients (55.5%). When given in a later therapy line, altogether 222 treatments (in 122 patients) were administered prior to first venetoclax with chemoimmunotherapy (70.3%) and chemotherapy (10.4%) being the most frequent used prior regimen (Figure 1a). Following the first documented treatment with venetoclax, most patients (90.5%) did not receive a subsequent therapy and in the remaining 9.5% of patients, up to 5 subsequent treatments were administered. These comprised 37 different subsequent treatments in 26 patients, of which 10 (27.0%) were venetoclax‐containing, 13 (35.1%) BTKi‐containing and only one was a triple combination with an anti‐CD20 antibody, BTKi, and venetoclax (2.7%).

**FIGURE 2:**
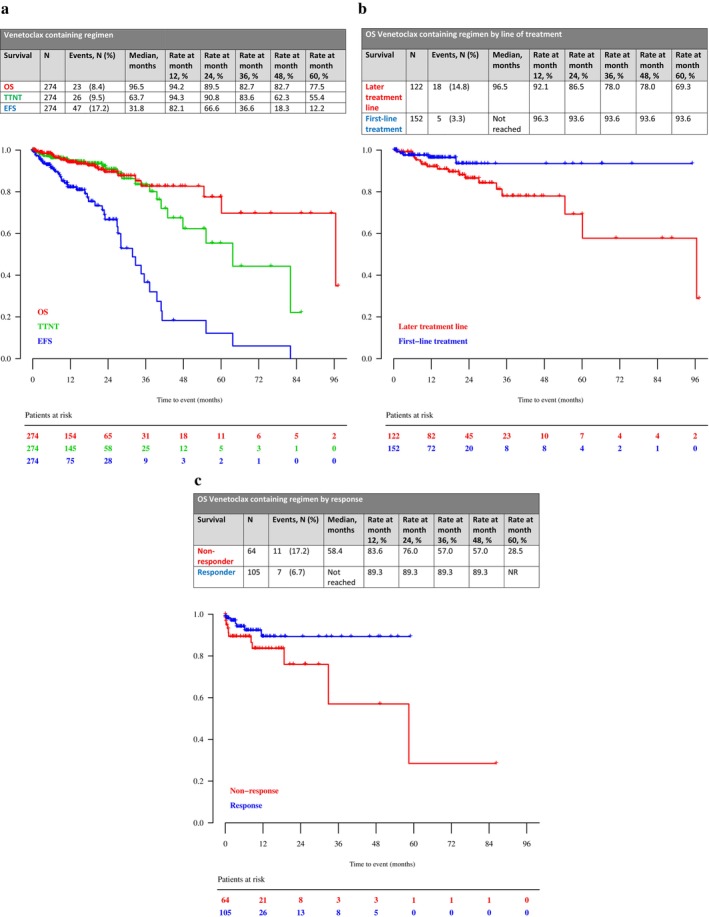
(a) OS, TTNT, EFS from start of first venetoclax containing regimen in the venetoclax cohort. (b) OS from start of first venetoclax containing regimen by line of venetoclax treatment in the venetoclax cohort. (c) OS from end of first venetoclax containing regimen by response in the venetoclax cohort.

The estimated 1‐year EFS rate from the start of the first documented venetoclax treatment was 82.1%, the estimated 1‐year TTNT rate was 94.3%, and the estimated 1‐year OS rate was 94.2% (Figure 2a). Twenty‐tree patients died, with most commonly reported cases of death being disease progressions and infections (21.7% each). The estimated 1‐year OS rate was 96.3% when venetoclax was given as first‐line treatment versus 92.1% when given for the first time in a later therapy line (HR 0.38, 95% CI 0.14–1.05, Figure 2b). The estimated 1‐year OS rate from end of first documented venetoclax treatment for therapy responders with a complete or partial remission was 89.3% and 83.6% for non‐responders (HR 0.41, 95% CI 0.16–1.07, Figure 2c).

In the BTKi cohort, median age at time of first BTKi treatment was 72 (range 32–92), with 610 male patients (68.7%). Median CIRS score was 3 (range 0–17) and 45 patients (9.6%) had an ECOG performance status > 1. Among 156 patients with available data, 104 patients (66.7%) had uIGHV and 61 out of 130 patients (46.9%) a deletion and/or mutation *TP53*. According to the CLL‐IPI, 46 out of 142 patients (32.4%) were in the very high‐risk group (Table 1).

Patients in the BTKi cohort received a median number of one prior treatment (range 0–12) before receiving BTKi for the first time and 351 of 888 patients (39.5%) received a BTKi as first‐line treatment. Altogether 1042 treatments (in 537 patients) were given prior to first BTKi. Chemoimmunotherapy (68.4%) and chemotherapy (17.9%) were the most frequent used prior regimen (Figure 1b). Most patients (71.6%) did not receive a subsequent therapy after BTKi treatment and up to 7 subsequent treatments were administered in 28.4% of the patients after first BTKi treatment. Altogether 377 subsequent treatments were documented in 252 patients, of which 140 (37.1%) were containing venetoclax, 99 (26.3%) a BTKi and only one was a triple combination with anti‐CD20 antibody, BTKi, and venetoclax (0.3%).

**FIGURE 3:**
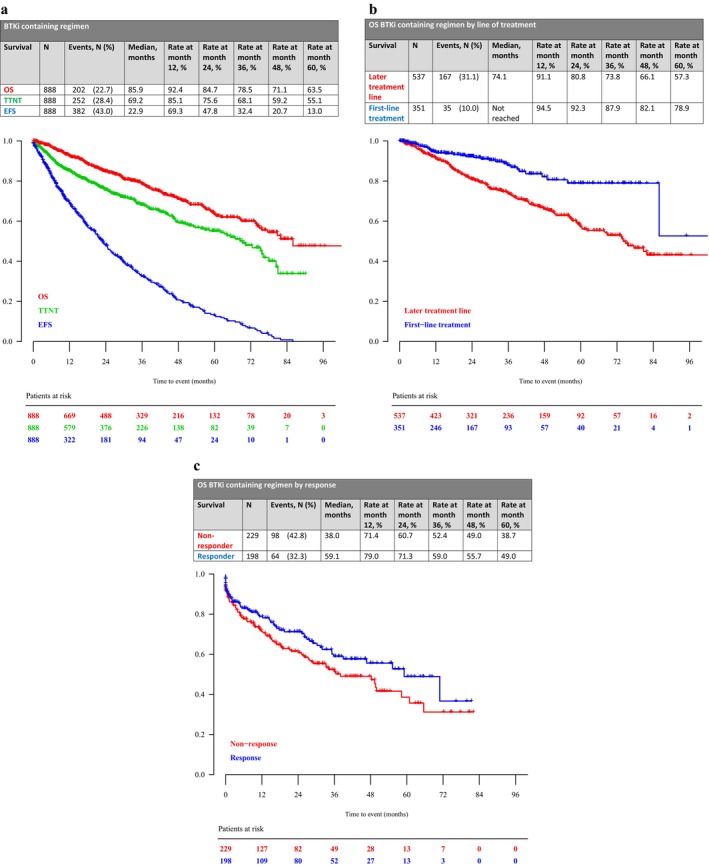
(a) OS, TTNT, EFS from start of first BTKi containing regimen in the BTKi cohort. (b) OS from start of first BTKi containing regimen by line of BTKi treatment in the BTKi cohort. (c) OS from end of first BTKi containing regimen by response in the BTKi cohort.

The estimated 3‐year EFS rate from start of first documented BTKi treatment was 32.4%, the estimated 3‐year TTNT rate was 68.1% and the estimated 3‐year OS rate was 78.5% (Figure 3a). Among 202 patients who died, the most commonly reported cases of death were disease progressions (27.0%) and infections (19.5%). When a BTKi was given as first‐line treatment, the estimated 3‐year OS rate was 87.9% compared to 73.8% when administered in a later therapy line (HR 0.43, 95% CI 0.30–0.61, Figure 3b). The estimated 3‐year OS rate from end of first documented BTKi of responders was 59.0% compared to 52.4% in non‐responders (HR 0.74, 95% CI 0.54–1.02, Figure 3c).

Differentiating further according to BTKi agents, 809 patients received their first BTKi treatment with ibrutinib and 79 with acalabrutinib. Median observation times from first treatment with either the first‐generation BTKi ibrutinib or the second‐generation BTKi acalabrutinib were 37 (range 0–120) and 9 months (range 0–62), the estimated 1‐year EFS rates from first BTKi treatment were 69.5% and 70.3%, the estimated 1‐year TTNT rates were 84.8% and 92.0%, and the 1‐year OS rates were 92.4% and 92.2%, respectively.

**FIGURE 4:**
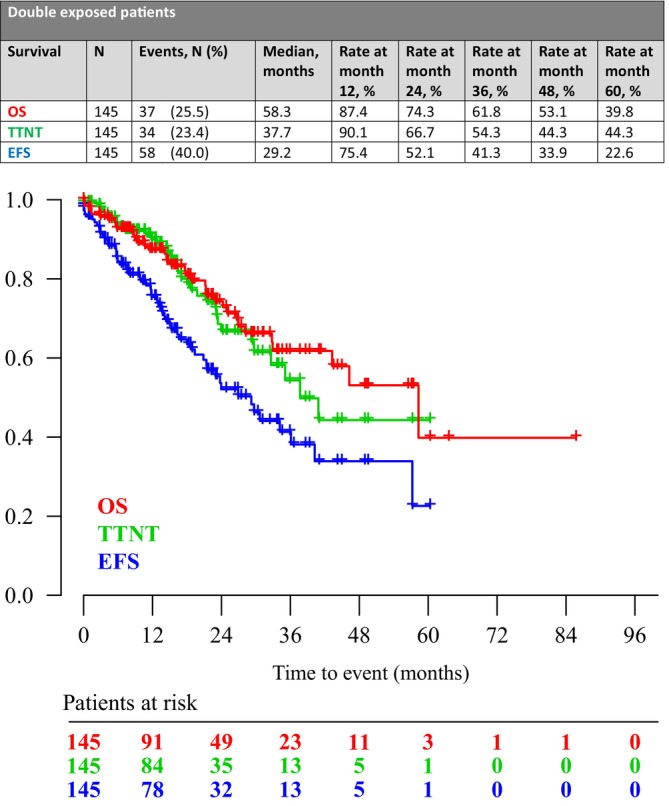
OS, TTNT, EFS from start of subsequent BTKi/Ven in the double exposed cohort.

A total of 145 patients were double exposed patients who had been treated with both BCL2i and BTKi. Of those, 12 patients have received venetoclax prior to BTKi, and 133 have been treated with a BTKi prior to venetoclax. The subgroup of double exposed patients had an estimated 1‐year EFS rate from subsequent treatment with either BTKi or venetoclax of 75.4%, an estimated 1‐year TTNT rate of 90.1%, and an estimated 1‐year OS rate of 87.4% (Figure 4a).

In summary, we here present data of two very heterogeneous patient cohorts from the GCLLSG registry, treated with either venetoclax or a BTKi as first administered targeted substance class within the documented treatment sequence, in order to give an outlook on the transition of these substances into clinical routine.

Baseline characteristics of the two cohorts seem to be comparable, but *TP53* aberrations were more frequent in the BTKi group, potentially uncovering treatment selection criteria used in clinical practice that favor the use of BTKi due to study data showing improved outcomes [11, 12, 13]. However, the imbalance in genetic risk factors should be interpreted with caution, as data were unavailable for many patients, which is mostly because they have not been analyzed by the treating physician in clinical routine. Different time points of EMA approval might also have affected observation times, treatment sequencing, prior treatment exposure, and patient characteristics. Nevertheless, EFS, TTNT, and OS seem to be comparable in both cohorts.

The results of the analyses seem to underline the efficacy of both BCL2i‐ and BTKi‐based regimens and suggest better survival outcomes when these agents are used as first‐line therapy. However, we are reporting real world data descriptively here and this includes missing data and sub‐standard management of the patients, which limits the interpretation of our results. Caveats to be considered are that outcomes on efficacy might be impacted by genetic high‐risk factors, which unfortunately have not been reported in many cases, but also by the choice of prior treatment regimen. Additionally, patients treated in later therapy lines often exhibit less favorable characteristics regarding the underlying disease and general condition, such as poorer performance status or organ function, and higher burden of comorbidities or frailty. Another observation is that the approved targeted substances translate only time‐delayed into clinical practice: a large proportion of patients were treated with chemo(immuno)therapy in advance despite the availability of targeted agents at that time. Several factors might have contributed to this delay, including the slow translation from evidence to clinical practice and hesitancy to use novel drugs with unfamiliar side effect profiles. A similar observation was made in a retrospective study in the era of chemoimmunotherapy [14].

The increasingly frequent use of targeted therapies reflects the evolving treatment paradigm, emphasizing the need for individualized therapy selection based on patient‐specific risk factors and molecular profiles [15]. Therefore, it is important to raise awareness that genetic risk factors should be determined before initiation of each new treatment line also outside of clinical trials in order to improve treatment decisions and enable a more comprehensive assessment of efficacy in real‐world populations. This is particularly important because the outcome of double‐exposed patients was poor, and new concepts for this patient population are urgently needed. Further randomized controlled data are warranted to optimize treatment sequencing and to explore potential combinations that may further improve long‐term outcomes in routine clinical practice.

## Author Contributions

N.K. and A.F. contributed to the design, data acquisition, data analysis and interpretation. R.L. and S.R. contributed to the design, data analysis and interpretation. H.L., T.I., S.D., J.L., A.A., R.S., J.D., O.A, P.L., P.C., M.H. and B.E. contributed to the data acquisition and interpretation. All authors helped to write the manuscript and approved the final version for publication.

## Funding

The authors have nothing to report.

## Ethics Statement

The authors have nothing to report.

## Conflicts of Interest

N.K.: Research support: AstraZeneca; Honoraria: AbbVie, AstraZeneca, BMS, Kite/Gilead, Lilly; Advisory Board: AstraZeneca, BMS, Janssen; Travel grants: AbbVie, AstraZeneca, BeOne, Janssen, Lilly. S.R.: Honoraria: MSD. H.L.: Consultant or advisory board member: Amgen, Novartis; Honoraria: Mundipharma, Amgen; Travel Support: Servier, Novartis, Janssen, Abbvie, BMS. S.D.: Advisory Board: BeOne, Abbvie, Incyte. O.A.S.: Honoraria/Advisory Fees: AbbVie, Adaptive, Ascentage, AstraZeneca, BeOne, Genmab, Gilead, Hikma, Janssen, Lilly, Roche; Research grants: AbbVie, BeOne, Janssen, Roche‐Genentech. P.L: Research support: Janssen; Advisory Board: BeOne, Janssen, AstraZeneca, AbbVie; Travel support: BeOne, AbbVie, Janssen, Roche. P.C.: Research funding: Acerta, AstraZeneca, BeOne, F. Hoffmann‐LaRoche, Gilead, Janssen‐Cilag, Novartis; honoraria: AbbVie, AstraZeneca, BeOne, BMS, F. Hoffmann‐LaRoche, Janssen‐Cilag; Advisory boards: Acerta, AstraZeneca, BeOne, Janssen‐Cilag, Novartis; Travel support: AbbVie, AstraZeneca, BeiOne, F. Hoffmann LaRoche, Gilead, Janssen‐Cilag, Novo Nordisk. M.H. Consultant or advisory board member, honoraria and research support: AbbVie, Amgen, Celgene, F. Hoffmann‐LaRoche, Gilead, Janssen‐Cilag, Mundipharma. B.E.: Consultant or advisory board member, research support or travel support by AbbVie, AstraZeneca, Celgene, F. Hoffmann‐LaRoche, Gilead, Janssen‐Cilag. K.F.: Advisory Board: AstraZeneca, AbbVie; speaker honoraria: AbbVie, Roche; research funding: AbbVie, Roche. A.F.: Research funding: AstraZeneca and Celgene, Honoraria: AstraZeneca, Travel grants: AbbVie.

## Data Availability

The data that support the findings of this study are available on request from the corresponding author. The data are not publicly available due to privacy or ethical restrictions.
